# Long-Term Immunity and Antibody Response: Challenges for Developing Efficient COVID-19 Vaccines

**DOI:** 10.3390/antib11020035

**Published:** 2022-05-12

**Authors:** Mohammad Reza Sepand, Banafsheh Bigdelou, Jim Q. Ho, Mohammad Sharaf, Alexis J. Lannigan, Ian M. Sullivan, Alecsander P. da Silva, Leland O. Barrett, Scott McGoldrick, Yuvraj Lnu, Shannon E. Lynch, Jared M. Boisclair, Dakarai D. Barnard-Pratt, Steven Zanganeh

**Affiliations:** 1 Department of Bioengineering, University of Massachusetts Dartmouth, 285 Old Westport Road, North Dartmouth, MA 02747, USA; msepand@umassd.edu (M.R.S.); bbigdelou@umassd.edu (B.B.); alannigan@umassd.edu (A.J.L.); isullivan1@umassd.edu (I.M.S.); adasilva10@umassd.edu (A.P.d.S.); lbarrett1@umassd.edu (L.O.B.); smcgoldrick@umassd.edu (S.M.); ylnu1@umassd.edu (Y.L.); slynch2@umassd.edu (S.E.L.); jboisclair@umassd.edu (J.M.B.); dpratt2@umassd.edu (D.D.B.-P.); 2 Department of Medicine, Albert Einstein College of Medicine, Bronx, NY 10461, USA; jimho28@gmail.com; 3 Department of Chemical and Biomolecular Engineering, New York University, New York, NY 10012, USA; mo.sharaf@nyu.edu

**Keywords:** SARS-CoV-2, immunogenicity, vaccine, COVID-19, antibodies

## Abstract

Questions and concerns regarding the efficacy and immunogenicity of coronavirus disease 2019 (COVID-19) vaccines have plagued scientists since the BNT162b2 mRNA vaccine was introduced in late 2020. As a result, decisions about vaccine boosters based on breakthrough infection rates and the decline of antibody titers have commanded worldwide attention and research. COVID-19 patients have displayed continued severe acute respiratory syndrome coronavirus 2 (SARS-CoV-2)-spike-protein-specific antibodies and neutralizing antibodies in longitudinal studies; in addition, cytokine activation has been detected at early steps following SARS-CoV-2 infection. Epitopes that are highly reactive and can mediate long-term antibody responses have been identified at the spike and ORF1ab proteins. The N-terminal domain of the S1 and S2 subunits is the location of important SARS-CoV-2 spike protein epitopes. High sequence identity between earlier and newer variants of SARS-CoV-2 and different degrees of sequence homology among endemic human coronaviruses have been observed. Understanding the extent and duration of protective immunity is consequential for determining the course of the COVID-19 pandemic. Further knowledge of memory responses to different variants of SARS-CoV-2 is needed to improve the design of the vaccine.

## 1. Introduction

Millions of individuals have been infected with the newly discovered severe acute respiratory syndrome coronavirus 2 (SARS-CoV-2) since early 2020, resulting in the coronavirus disease 2019 (COVID-19) pandemic [1]. SARS-CoV-2, like SARS-CoV-1 and MERS-CoV, is mainly a respiratory virus that causes manifestations and illnesses ranging from minor infections to severe acute respiratory syndrome, leading to multiorgan failure and death in some cases. Various COVID-19 protection and management strategies, such as convalescent plasma, monoclonal antibodies, off-label drugs, and numerous vaccinations, using advances in materials science strategies for targeted delivery, are being investigated in reaction to the outbreak [1]. About 200 vaccine candidates are now being studied worldwide, but work on a harmless and highly immunogenic COVID-19 vaccine is still ongoing. Inability to produce long-term immunity and a failure to control the cytokine storm are serious roadblocks in improving and approving the COVID-19 vaccine [2]. Table 1 summarizes some of the benefits and drawbacks of various vaccination types.

For maximum protection, most licensed vaccinations in use and several vaccines still in progress appear to require two doses, which create logistical issues and limit availability (Table 2). Furthermore, the cold chain requirement, particularly ultra-cold chains for mRNA-based vaccines, obstructs vaccine distribution in impoverished countries [1]. The continuing evolution and successive virus mutations, which can considerably diminish vaccine-induced immunity, are further obstacles [11]. While no data of “antigenic drift” have been found, such as with the influenza virus, mutations can happen and vaccine-mediated immune selection pressure may speed the escape mutants’ rise, as has been documented with other infections [12,13]. Therefore, COVID-19 vaccine development must continue to be improved and researched.

## 2. COVID-19 Vaccine-Mediated Protection: Immunological Mechanisms

**Figure 1:**
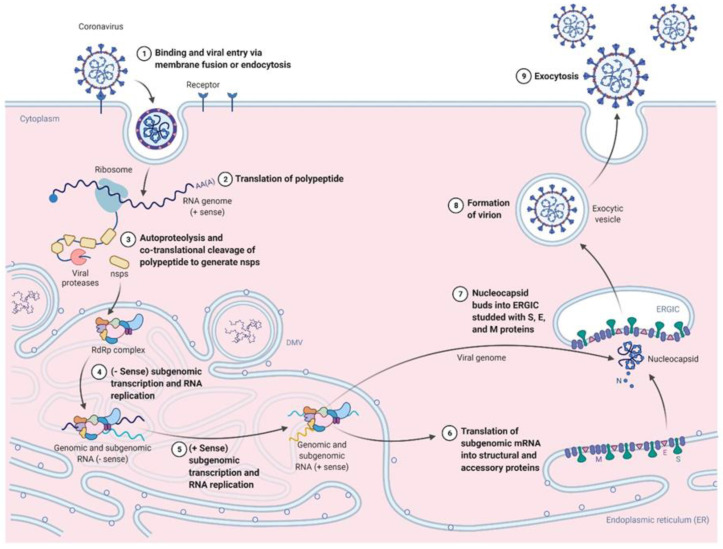
The SARS-CoV-2 life cycle.

SARS-CoV-2 is a single-stranded positive-sense RNA virus from the *Coronaviridae* family and the β-coronavirus genus [17]. A 79.6% nucleotide sequence parallel between SARS-CoV-1 and SARS-CoV-2 has been found using whole-genome sequencing techniques [18]. The SARS-CoV-2 genome encodes several structural proteins: the spike (S), envelope (E), membrane (M), and nucleocapsid (N) proteins. The S protein, a trimeric type I fusion protein found on the virion surface, is essential for viral adhesion and entrance into cells of the host. For viral infection, host proteases must cleave the S protein into 2 subunits; S1 and S2 [19]. Virus attachment to angiotensin-converting enzyme 2 (ACE2) as its entrance receptor is made through the virus’s S1 subunit receptor-binding domain (RBD) [17,20]. The S1 subunit gets released after its interaction with ACE2. Then, for viral fusion and entry, the fusion peptide of the S2 subunit is utilized (Figure 1) [21].

By activating adaptive immune responses, neutralizing antibodies could inhibit viral infection and eliminate its pathogens. Infected patients between 7 and 14 days following infection with SARS-CoV-2 revealed a high degree (more than 90%) of seroconversion [22,23]. The production of IgA, IgG, and IgM that targeted the spike and nucleocapsid proteins of SARS-CoV-2 were detected to occur during the acute and early recovery phases of viral infection [22,23]. Factors such as age, symptomatic infection, and severity of disease seem to influence the amount of neutralizing antibodies. Elderly individuals with COVID-19 appear to have poorer neutralizing antibody responses [24,25,26]. Although the plasma/serum reactivity to antigens of the virus and titers of neutralizing antibodies were reduced in certain people with symptomatic COVID-19 over time, several studies regarding the endurance of antibody responses have demonstrated that a continuous level of general long-term humoral immunity could be seen for as long as 8 to 12 months post-infection in COVID-19-convalescent patients [25,27]. In addition, the amount of SARS-CoV-2 antigen-specific memory B cells remained steady for 6 to 12 months at least [27], as well as continued B-cell clonal selection and accumulation with the release of neutralizing antibodies [28], demonstrating the continuance of lasting humoral immunity following infection by the coronavirus.

Neutralizing antibodies have a vital function in protecting the host from viral illness. Many highly effective SARS-CoV-2 monoclonal antibodies have been recognized, [29,30] principally directed at epitopes found at the RBD of the S protein, which collects into trimers on the surface of the virion and enables viral entry and fusion after activating the ACE2 receptor [31]. Other S protein regions, such as the S1 and S2 subunit N-terminal domains, include immunogenic epitopes that can engage neutralizing antibodies as well [30,32]. Effector pathways mediated by Fc receptors that interact with SARS-CoV-2 antibodies can also protect the host, such as the antibody-mediated cell toxicity and antibody-dependent cellular phagocytosis by natural killer cells and macrophages or monocytes, respectively [33,34]. As well as fighting viral infections, antibodies produced by either natural infection or via vaccination can assist viral development, via elevated activation of inflammation or by heightened virus infectivity (e.g., Fc-dependent or forming antigen/antibody immune complexes) [35,36]. However, such increased viral infection has not been seen in vivo for SARS-CoV-2 infection. These differing antibody responses require a thorough investigation of SARS-CoV-2 epitopes and characteristics of antibodies focused on neutralizing or non-neutralizing epitopes.

Both humoral and cellular immunity should be considered while developing COVID-19 vaccines. Because neutralizing antibodies primarily target the critical SARS-CoV-2 S protein epitopes, this protein is employed in developing vaccines against coronavirus infection [21,37,38,39]. Some antibodies can neutralize the virus by targeting the RBD and preventing viral attachment to surface receptors of host cells and consequently inhibit viral entry and invasion. The strong neutralizing ability of synthetic peptides and antibodies against the heptad repeat region, HR2, of SARS-CoV-1’s S2 subunit has been reported [40,41,42,43], most likely inhibiting the conformational modifications required for membrane fusion. Moreover, since targeting the S protein may efficiently induce the T-cell immune response, it is considered for vaccine development. As a result, attempts to produce a SARS-CoV-2 vaccine have induced responses to the S protein. N and M proteins have also been found to trigger an effective cellular immune response in the body [44]. Clinical trials are currently underway to test vaccines using N proteins (NCT04591717 and NCT04715997).

In addition to innate immune responses, the capability of mRNA and adenovirus (AdV) vaccines to boost intracellular S protein synthesis should activate both CD8+ and CD4+ T cells to grow into functional and memory subtypes [2]. After vaccination, CD4+ and CD8+ effector T cells develop in response to type I interferon, which regulates leukocytes and inflammation, including the stimulation of CD4+ T follicular helper (TFH) cells. This in turn allows the differentiation of B cells into plasma cells that secrete antibodies. Two primary doses of the mRNA and AdV vaccines separated by 3 to 4 weeks have been recommended. Mild to moderate adverse effects are associated with the vaccines, including transient fever, chills, and pain at the injection site. Elevated secondary immune responses may be due to “trained immunity”, which affects the innate immune response to create immunological memory [45], or mobilization of memory B and T cells from a previous exposure. Because the development and survival of B cells and T cell memory can be induced by type I interferon, long-term immunological memory can be promoted by the inflammation from booster vaccines.

## 3. What Is the Duration of COVID-19 Immunity?

COVID-19 is a novel illness, and researchers are still trying to discover how the body will react to the vaccine. One of the main issues with the available COVID-19 vaccines is their ability to elicit strong and long-lasting immunity, and it is sometimes uncertain how long any protection will remain. Scientists believe that COVID-19 immunization could produce immunity that lasts for several months, based on our knowledge about other viruses and what we know about antibodies in COVID-19 patients and individuals who have been immunized. However, the lack of availability or use of the World Health Organization (WHO) International Standard across different labs and clinical trials makes it difficult to compare the data among them. Other factors such as T and B cell memory, which according to some research may continue for years, can impact immunity. Finally, because natural infection produces different immunity than vaccination, research cannot always be pooled to get a definitive conclusion.

For months after immunization, anti-spike protein IgG antibodies and other neutralizing antibodies specific to the virus are produced [46,47]. However, T cell evidence is still being clarified. This short-term durability will likely be enough to stop the SARS-CoV-2 spread and recover. Nevertheless, the worldwide spread of SARS-CoV-2 and the appearance of S protein variations may restrict vaccination effectiveness. Because of reservoirs in unvaccinated persons and/or other species of animals, eradicating SARS-CoV-2 from people could be challenging. SARS-CoV-2 vaccines can be provided once or twice per year for persistent strains or seasonal variants. New SARS-CoV-2 vaccines can be created from variant spike protein sequences and other proteins. The formulation of the mRNA vaccine is suitable for repeated or revamped vaccination because various mRNAs, including mutant S proteins, can be readily produced and placed inside the lipid nanoparticle (LNP) carrier. Due to immune-mediated vector clearance, the AdV vector formulation results in AdV-specific immunity, limiting the efficiency of repeating boosters [2].

Variation in vaccine reactions will undoubtedly emerge due to the enormous volume and simultaneous vaccination of the entire world’s people, with some persons failing to produce significant antibody responses or being protected. Tissue-resident memory T (T_RM_) cells can offer immunity against respiratory viruses because they are formed in the lungs after the first infection and facilitate protective responses in situ upon viral re-challenge [48]. T_RM_ cells can be stimulated using attenuated viral vaccine formulations and site-specific immunization. [48]. It would be fascinating to define whether mRNA vaccines’ intranasal delivery can stimulate T_RM_ cells and lung protection. A recent study showed that an intranasal vaccine provided strong immunological protection against SARS-CoV-2 infection in the respiratory tract of mice [49,50]. Self-replicating mRNA vaccines (which imitate replication of viruses) can promote protective T cell immunity in a similar way. Vaccine composition and delivery mode adjustments can be used to tailor vaccinations to different immunological states and ages.

The SARS-CoV-2 B.1 and D614G variants have been the focus of most research. Whether re-infection can be prevented in other SARS-CoV-2 variants (e.g., B1.1.7, B.1.351, P.1) due to the humoral response is unclear. It is currently unclear whether modified vaccines targeting new SARS-CoV-2 variants are superior to the original vaccines. Preliminary data from small preclinical studies suggested that Omicron-specific vaccines did not perform better than the original vaccines [51].

Furthermore, new research demonstrates that the induction of trained immunity by the Bacillus Calmette–Guérin (BCG) vaccine provides significant protection against various viral diseases [52]. BCG is a live attenuated tuberculosis vaccine created at the Pasteur Institute in Paris at the turn of the twentieth century. BCG-enabled trained immunity may protect against COVID-19; however, this theory must be tested in randomized clinical trials. The BRACE trial is currently investigating the use of the BCG vaccine against COVID-19 in healthcare workers (NCT04327206). In addition, there are other potential strategies to stimulate trained immunity in COVID-19. One example is the oral polio vaccine, which may provide protection for other viral infections. Another example is VPM1002, which is a novel recombinant BCG vaccine.

## 4. Persistence of SARS-CoV-2 Antibodies in the Body

Research indicates that neutralizing antibodies persist for months in individuals with COVID-19 but that the amount of antibodies gradually decreases over time. In a study of 5882 recovered patients, antibodies in the blood of patients with mild to severe infection were documented from five to seven months post-onset [53]. However, patients with severe infection demonstrated more antibodies overall. In June 2021, the Moderna vaccine research group reported that, six months following their second dose, clinical trial participants were found to have high antibody levels [54]. In March 2022, another study reported that a Moderna vaccine booster after a Pfizer–BioNTech or AstraZeneca primary immunization provided higher protection against the Omicron variant (observed 2 to 4 weeks post-booster), but the protection decreased over time (by 5 to 9 weeks post-booster) [55]. Another investigation clarified that a low dose Moderna vaccine could elicit long-lasting antibodies, memory CD4^+^ T cells including TFH and IFNγ-expressing cells, and CD8^+^ T cells, enhancing cellular and humoral immunity. Finally, a new investigation reported the Oxford–AstraZeneca vaccine led to high levels of antibodies after a single dose, with “minimal waning” three months later [56].

Dufloo et al., investigated amounts and functions of antibodies in symptomatic or asymptomatic patients [57]. Both groups of patients exhibited polyfunctional antibodies, that can counteract the virus or help kill virus-infected cells. The study suggests that this broad reaction may help promote longer protection in general, even if neutralizing potential decreases. Moreover, another study explored the decline of neutralizing antibodies in seven COVID-19 vaccines [58]. Researchers believe even in the absence of the booster dose, long-term immunity of considerable people proportion kept against severe infection by an antigenically identical strain, despite susceptibility to mild disease [58].

## 5. Factors Involved in Inducing Long-Term Immunity for COVID-19 Vaccines

The loss of short-lived plasma cells is thought to be the reason for the first rapid decline in antibodies in SARS-CoV-2, whereas the long-lived plasma cells’ development is thought to be the cause of the plateau in antibody response [59]. Kaneko et al., studied the underlying causes of waning and observed the lack of germinal centers in the thoracic lymph nodes of dead SARS-CoV-2 cases [60]. They suggested that Bcl6+ follicular T-cell defects that result in loss of their ability in memory B-cell (MBC) activation are conferred in this germinal center absence. This would decrease the production of long-lasting, high-affinity antibodies, which might also elucidate why antibodies to SARS-CoV-2 are rapidly fading. MBCs are assumed to be kept independently of antibody levels, implying that B-cell immunity could persist even if antibody levels decline. However, because only deceased cases were examined, these pathways may only pertain to the most severe illness.

Antibodies are identified to play a vital function in the immune system. Deeper understanding of the epitope landscape of antibodies to SARS-CoV-2, particularly S protein–directed epitopes, offers novel wisdom that enables innovative and sound vaccine design. Some studies suggest that antibodies are not enough to eliminate viruses, which is confirmed by the lack of a sharp drop in viral load post-seroconversion [61]. Monitoring the clinical outcome of convalescent plasma transfer therapy (CPTT) may be one approach to determining antibody efficacy [62]. CPTT trials in SARS-CoV-2 have yielded mixed results on the preventive role of neutralizing antibodies. In case–control trials for severe SARS-CoV-2, CPTT initially showed encouraging results, with some research suggesting that earlier treatment was more effective [62,63]. SARS-CoV-1 patients were also found to benefit from early CPTT [64]. Recent findings from large-scale randomized controlled studies, on the other hand, found no significant reduction in mortality or benefits in clinical outcomes for patients with moderate or severe SARS-CoV-2 who were given CPTT [65]. As a result, the antibody response alone may not be as crucial in SARS-CoV-2 protection as previously believed.

Vaccine acceptability, responses, and effectiveness have been documented to be influenced by sex and gender (biological and cultural differences, respectively) [66]. Females are less likely than males to receive vaccines, but once vaccinated, they generate greater and longer-lasting protective antibody responses [66]. Females might, however, experience adverse reactions more frequently and intensely than males who are vaccinated [66]. Sex hormone control of immune function, and genetic and epigenetic modulation, are among the processes explaining sex disparities in vaccine response. One possible reason for the stronger and sometimes longer-lasting immunological reactions in women is that they have two X chromosomes, whereas males have only one [67,68,69]. Since the X chromosome contains many genes involved in the immune system (e.g., toll-like receptor 7 [TLR7], chemokines, interleukins, microRNAs), females may express more molecules that participate in the immune response than males.

Another parameter that influences immune response modulation is age, which should not be overlooked. A study on the elderly found delayed and decreased T-cell and antibody responses following BNT162b2 vaccination [70]. Another study on SARS-CoV-2–naive nursing home residents also found poor antibody responses to BNT162b2 vaccination [71]. In this sense, the immune system’s ability to function declines as people age [72]. An abnormal, chronic, low-grade pro-inflammatory condition, which may occur at a larger level in females than in males, is a prominent characteristic of the immune system in aging [73]. Furthermore, Wang et al., found that influenza vaccination is linked to lower hospitalization and fatality rates in older women than in men, implying that females have higher titers of the hemagglutination inhibition (HAI) test or react more strongly to vaccination, resulting in better defense than men [74].

## 6. Concerns about Boosting COVID-19 Vaccines

A COVID-19 infection wave produced by the extremely transmissible Delta variant increased the global public health emergency, prompting researchers to evaluate whether booster doses for vaccinated people were necessary and when they should be given [75]. Boosting may be appropriate for some persons whose first immunization, defined as the initial one-dose or two-dose series of each vaccine, did not give adequate safety, such as those given low-efficacy vaccines or who are immunocompromised [76]. However, persons who had a poor response to the original vaccine may also have a poor response to a booster. It is uncertain if an extra dose of the same vaccine or a distinct vaccine that complements the first immune response will benefit people with compromised immune systems. Because the immune response gradually decreases after the initial vaccination and variants with novel antigens may no longer elicit a sufficient immune response, booster vaccines are recommended.

Research in Israel just after booster dosages were allowed and broadly utilized found that a booster dose could reduce the rates of infection and severe illness compared with no booster [77]. The long-term effect of boosters on clinical outcomes should be further investigated. Large numbers of adults in the United States are completely vaccinated, whereas others are not, and comprehensive comparisons between them are continuing. New data from sizeable studies in the United States (COVID-NET and large health maintenance organizations [78,79]) show that complete immunization against serious disease or hospitalization continues to be highly effective.

## 7. Conclusions and Future Prospects

COVID-19 vaccines that have been authorized or are in development are predicted to give at least some immunity against novel variations because they elicit a comprehensive immune response involving a variety of antibodies and cells. However, the longevity of such an immunological response to SARS-CoV-2 vaccination is still unknown. Natural coronavirus-induced antibody titers have been shown to fade with time. According to documented occurrences of SARS-CoV-2 reinfection and data of short-lived immunity to endemic human coronavirus infections, protective immunity following coronavirus infection could last months rather than years. The new variant of SARS-CoV-2 is more transmissible than previous variants. According to specific reports, existing COVID-19 vaccinations may be less effective in preventing infection with mutations. Furthermore, recent evidence suggests that the immunity afforded by COVID-19 vaccines wanes with time, making vaccinated people more vulnerable to infection with the Delta and Omicron variants of SARS-CoV-2. A booster shot of some of the most common COVID-19 vaccines may supply additional protection by inducing antibody-producing B cells to proliferate and raise antibody levels against the pathogen once more.

Multiple delivery methods should be investigated since they can be developed to distribute antigen and adjuvant payloads and contribute to multivalent antigen presentation, resulting in more effective vaccinations with improved immune reactions. Advances in nanotechnology, in particular, have opened up new avenues for vaccine development, allowing for the rapid development of novel candidate vaccine formulations. Because their contents are shielded against premature breakdown and unexpected targets such as macrophages, nanoparticle vaccine formulations are more effective than molecular vaccines solely [80]. Furthermore, lyophilized nanoparticles do not require refrigeration for storage. When targeting moieties are put on the surface of nanoparticle-based formulations, they may raise uptake by phagocytic APCs. Due to adjuvant co-delivery, APC-targeting nanoparticles are great vaccines for efficiently and appropriately training T and B cells, along with inducing high levels of antibody synthesis [81,82,83,84].

## Figures and Tables

**Table 1:** Some of the benefits and drawbacks of various vaccine types.

Vaccine Types	Mechanisms	Benefits	Drawbacks	References
DNA vaccines (e.g., Inovio INO-4800)	-The DNA can penetrate the cell and produce target antigens through the host cell’s transcription and translation systems.	-Live viruses are not required for DNA vaccinations. -Plasmid DNA is made relatively easily, and compared with the viruses, double-stranded DNA molecules are more stable and, for storing long-term, can be freeze-dried.	-Their application may be limited due to the delivery method. It is challenging for the administered vaccine to reach the cell nucleus. Physical delivery methods are usually needed (e.g., electroporation). -They have the potential to integrate into the genome.	[3,4,5,6]
mRNA vaccines (e.g., Moderna mRNA-1273 and Pfizer-BioNTech BNT162b2)	-The mRNA can enter the cytoplasm and undergo translation in the host cells.	-Theoretically, they are safer since they do not directly interact with the host DNA.	-The vaccines’ immunological effect may be insufficient, and the vaccines are often delivered as part of a complex to increase efficacy. -They may need lower storage temperature. -Vaccine efficacy may be reduced by activation of an antiviral immune response involving interferons.	[3,4,7,8]
Non-replicating viral vector vaccines (e.g., Johnson & Johnson JNJ-78436735, Oxford-AstraZeneca ChAdOx1, Sputnik V Gam-COVID-Vac)	-Viral vectors can encode the antigens of interest in a different virus. The antigens mimic an infection in the body.	-The efficacy of these vaccines is fairly high. -They have been studied for decades.	-The manufacturing process is relatively complex. -Recombinant viruses have the ability to integrate into the host genome. -The body may have pre-existing immunity to the vector, which can decrease the immune response.	[3,4,9,10]
Inactivated vaccines (e.g., Sinopharm BBIBP-CorV and Sinovac CoronaVac)	-These vaccines can be created using chemical and radiation inactivation techniques, which result in the loss of viral pathogenicity.	-They can elicit strong immune responses. -They have been widely studied and are known to be relatively safe.	-The immunogenic epitopes may be altered during inactivation. -Inactivated vaccines for SARS coronavirus have been associated with lung pathology involving eosinophils.	[3,10]
Live attenuated vaccines	-These vaccines have diminished pathogenicity of the virus through mutations or deletions in the viral genome.	-They mimic natural infections and have high immunogenicity.	-They could still be pathogenic in the host, especially in the immunocompromised.	[4,10]
Subunit vaccines (e.g., Novavax NVX-CoV2373)	-They use fragments of viral antigens created from recombinant protein methods to elicit an immune response.	-They have a high level of safety. -They are relatively simple to produce.	-They have relatively lower immunogenicity. Thus, they are often used with adjuvants.	[4,10]

**Table 2:** Specific characteristics of some COVID-19 vaccines in use.

Vaccine Name	Vaccine Type [10]	Platform	Original Dose	Storage Temperature [14,15,16]
Moderna (mRNA-1273)	mRNA vaccine	mRNA in lipid nanoparticle	2	−50 °C to −15 °C (up to 9 months) 2° to 8 °C (up to 30 days)
Pfizer-BioNTech (BNT162b2)	mRNA vaccine	mRNA in lipid nanoparticle	2	Formulation for 12 years or older: −90 °C to −60 °C (up to 9 months) −25 °C to −15 °C (up to 2 weeks) 2 °C to 8 °C (up to 31 days after thawing)
Johnson & Johnson (JNJ-78436735)	Non-replicating viral vector vaccine	Non-replicating human adenovirus	1	2 °C to 8 °C
Oxford–AstraZeneca (ChAdOx1)	Non-replicating viral vector vaccine	Non-replicating chimpanzee adenovirus	2	2 °C to 8 °C
Sputnik V (Gam-COVID-Vac)	Non-replicating viral vector vaccine	Non-replicating human adenovirus	2	−18 °C
Sinopharm (BBIBP-CorV)	Inactivated vaccine	Inactivated virus	2	2 °C to 8 °C
Sinovac (CoronaVac)	Inactivated vaccine	Inactivated virus	2	2 °C to 8 °C
Novavax (NVX-CoV2373)	Subunit vaccine	Spike protein and Matrix M adjuvant	2	2 °C to 8 °C

## Data Availability

No new data were created or analyzed in this study. Data sharing is not applicable to this article.

## References

[1] Bok K., Sitar S., Graham B.S., Mascola J.R. (2021). Accelerated COVID-19 vaccine development: Milestones, lessons, and prospects. Immunity.

[2] Teijaro J.R., Farber D.L. (2021). COVID-19 vaccines: Modes of immune activation and future challenges. Nat. Rev. Immunol..

[3] Li Y.-D., Chi W.-Y., Su J.-H., Ferrall L., Hung C.-F., Wu T.-C. (2020). Coronavirus vaccine development: From SARS and MERS to COVID-19. J. Biomed. Sci..

[4] Han X., Xu P., Ye Q. (2021). Analysis of COVID-19 vaccines: Types, thoughts, and application. J. Clin. Lab. Anal..

[5] Suschak J.J., Williams J.A., Schmaljohn C.S. (2017). Advancements in DNA vaccine vectors, non-mechanical delivery methods, and molecular adjuvants to increase immunogenicity. Hum. Vaccines Immunother..

[6] Blakney A.K., Bekker L.-G. (2022). DNA vaccines join the fight against COVID-19. Lancet.

[7] Rahman M.A., Islam M.S. (2021). Early approval of COVID-19 vaccines: Pros and cons. Hum. Vaccines Immunother..

[8] Chaudhary N., Weissman D., Whitehead K.A. (2021). mRNA vaccines for infectious diseases: Principles, delivery and clinical translation. Nat. Rev. Drug Discov..

[9] Kremer E.J. (2020). Pros and cons of adenovirus-based SARS-CoV-2 vaccines. Mol. Ther..

[10] Kyriakidis N.C., López-Cortés A., González E.V., Grimaldos A.B., Prado E.O. (2021). SARS-CoV-2 vaccines strategies: A comprehensive review of phase 3 candidates. Npj Vaccines.

[11] Kimman T.G., Cornelissen L.A., Moormann R.J., Rebel J.M., Stockhofe-Zurwieden N. (2009). Challenges for porcine reproductive and respiratory syndrome virus (PRRSV) vaccinology. Vaccine.

[12] Altman M.O., Angeletti D., Yewdell J.W. (2018). Antibody immunodominance: The key to understanding influenza virus antigenic drift. Viral Immunol..

[13] Ning T., Nie J., Huang W., Li C., Li X., Liu Q., Zhao H., Wang Y. (2019). Antigenic drift of influenza A (H7N9) virus hemagglutinin. J. Infect. Dis..

[14] Nadimuthu L.P.R., Victor K. (2022). Environmental friendly micro cold storage for last-mile Covid-19 vaccine logistics. Environ. Sci. Pollut. Res..

[15] Santos A.F., Gaspar P.D., Souza H.J.D. (2021). Refrigeration of COVID-19 vaccines: Ideal storage characteristics, energy efficiency and environmental impacts of various vaccine options. Energies.

[16] Mercer J., Liang A., Yoon J., Nguyen J., Carroll J., Coley K.C. (2022). COVID-19 pandemic vaccination preparedness strategies for independent community pharmacies. J. Am. Pharm. Assoc.

[17] Zhou P., Yang X.-L., Wang X.-G., Hu B., Zhang L., Zhang W., Si H.-R., Zhu Y., Li B., Huang C.-L. (2020). A pneumonia outbreak associated with a new coronavirus of probable bat origin. Nature.

[18] Wu F., Zhao S., Yu B., Chen Y.-M., Wang W., Song Z.-G., Hu Y., Tao Z.-W., Tian J.-H., Pei Y.-Y. (2020). A new coronavirus associated with human respiratory disease in China. Nature.

[19] Jaimes J.A., Millet J.K., Whittaker G.R. (2020). Proteolytic cleavage of the SARS-CoV-2 spike protein and the role of the novel S1/S2 site. IScience.

[20] Hoffmann M., Kleine-Weber H., Schroeder S., Krüger N., Herrler T., Erichsen S., Schiergens T.S., Herrler G., Wu N.-H., Nitsche A. (2020). SARS-CoV-2 cell entry depends on ACE2 and TMPRSS2 and is blocked by a clinically proven protease inhibitor. Cell.

[21] Du L., He Y., Zhou Y., Liu S., Zheng B.-J., Jiang S. (2009). The spike protein of SARS-CoV—a target for vaccine and therapeutic development. Nat. Rev. Microbiol..

[22] Moderbacher C.R., Ramirez S.I., Dan J.M., Grifoni A., Hastie K.M., Weiskopf D., Belanger S., Abbott R.K., Kim C., Choi J. (2020). Antigen-specific adaptive immunity to SARS-CoV-2 in acute COVID-19 and associations with age and disease severity. Cell.

[23] Wu J., Liang B., Chen C., Wang H., Fang Y., Shen S., Yang X., Wang B., Chen L., Chen Q. (2021). SARS-CoV-2 infection induces sustained humoral immune responses in convalescent patients following symptomatic COVID-19. Nat. Commun..

[24] Zhang J., Wu Q., Liu Z., Wang Q., Wu J., Hu Y., Bai T., Xie T., Huang M., Wu T. (2021). Spike-specific circulating T follicular helper cell and cross-neutralizing antibody responses in COVID-19-convalescent individuals. Nat. Microbiol..

[25] Vanshylla K., Di Cristanziano V., Kleipass F., Dewald F., Schommers P., Gieselmann L., Gruell H., Schlotz M., Ercanoglu M.S., Stumpf R. (2021). Kinetics and correlates of the neutralizing antibody response to SARS-CoV-2 infection in humans. Cell Host Microbe.

[26] Bajaj V., Gadi N., Spihlman A.P., Wu S.C., Choi C.H., Moulton V.R. (2021). Aging, immunity, and COVID-19: How age influences the host immune response to coronavirus infections?. Front. Physiol..

[27] Dan J.M., Mateus J., Kato Y., Hastie K.M., Yu E.D., Faliti C.E., Grifoni A., Ramirez S.I., Haupt S., Frazier A. (2021). Immunological memory to SARS-CoV-2 assessed for up to 8 months after infection. Science.

[28] Sokal A., Chappert P., Barba-Spaeth G., Roeser A., Fourati S., Azzaoui I., Vandenberghe A., Fernandez I., Meola A., Bouvier-Alias M. (2021). Maturation and persistence of the anti-SARS-CoV-2 memory B cell response. Cell.

[29] Ju B., Zhang Q., Ge J., Wang R., Sun J., Ge X., Yu J., Shan S., Zhou B., Song S. (2020). Human neutralizing antibodies elicited by SARS-CoV-2 infection. Nature.

[30] Liu L., Wang P., Nair M.S., Yu J., Rapp M., Wang Q., Luo Y., Chan J.F.-W., Sahi V., Figueroa A. (2020). Potent neutralizing antibodies against multiple epitopes on SARS-CoV-2 spike. Nature.

[31] Xu C., Wang Y., Liu C., Zhang C., Han W., Hong X., Wang Y., Hong Q., Wang S., Zhao Q. (2021). Conformational dynamics of SARS-CoV-2 trimeric spike glycoprotein in complex with receptor ACE2 revealed by cryo-EM. Sci. Adv..

[32] McCallum M., De Marco A., Lempp F.A., Tortorici M.A., Pinto D., Walls A.C., Beltramello M., Chen A., Liu Z., Zatta F. (2021). N-terminal domain antigenic mapping reveals a site of vulnerability for SARS-CoV-2. Cell.

[33] Tortorici M.A., Beltramello M., Lempp F.A., Pinto D., Dang H.V., Rosen L.E., McCallum M., Bowen J., Minola A., Jaconi S. (2020). Ultrapotent human antibodies protect against SARS-CoV-2 challenge via multiple mechanisms. Science.

[34] Pinto D., Park Y.-J., Beltramello M., Walls A.C., Tortorici M.A., Bianchi S., Jaconi S., Culap K., Zatta F., De Marco A. (2020). Cross-neutralization of SARS-CoV-2 by a human monoclonal SARS-CoV antibody. Nature.

[35] Liu L., Wei Q., Lin Q., Fang J., Wang H., Kwok H., Tang H., Nishiura K., Peng J., Tan Z. (2019). Anti–spike IgG causes severe acute lung injury by skewing macrophage responses during acute SARS-CoV infection. JCI Insight.

[36] Li D., Edwards R.J., Manne K., Martinez D.R., Schäfer A., Alam S.M., Wiehe K., Lu X., Parks R., Sutherland L.L. (2021). In vitro and in vivo functions of SARS-CoV-2 infection-enhancing and neutralizing antibodies. Cell.

[37] Escriou N., Callendret B., Lorin V., Combredet C., Marianneau P., Février M., Tangy F. (2014). Protection from SARS coronavirus conferred by live measles vaccine expressing the spike glycoprotein. Virology.

[38] Liniger M., Zuniga A., Tamin A., Azzouz-Morin T.N., Knuchel M., Marty R.R., Wiegand M., Weibel S., Kelvin D., Rota P.A. (2008). Induction of neutralising antibodies and cellular immune responses against SARS coronavirus by recombinant measles viruses. Vaccine.

[39] Zhu F.-C., Li Y.-H., Guan X.-H., Hou L.-H., Wang W.-J., Li J.-X., Wu S.-P., Wang B.-S., Wang Z., Wang L. (2020). Safety, tolerability, and immunogenicity of a recombinant adenovirus type-5 vectored COVID-19 vaccine: A dose-escalation, open-label, non-randomised, first-in-human trial. Lancet.

[40] Bosch B.J., Martina B.E., Van Der Zee R., Lepault J., Haijema B.J., Versluis C., Heck A.J., De Groot R., Osterhaus A.D., Rottier P.J. (2004). Severe acute respiratory syndrome coronavirus (SARS-CoV) infection inhibition using spike protein heptad repeat-derived peptides. Proc. Natl. Acad. Sci. USA.

[41] Keng C.-T., Zhang A., Shen S., Lip K.-M., Fielding B.C., Tan T.H., Chou C.-F., Loh C.B., Wang S., Fu J. (2005). Amino acids 1055 to 1192 in the S2 region of severe acute respiratory syndrome coronavirus S protein induce neutralizing antibodies: Implications for the development of vaccines and antiviral agents. J. Virol..

[42] Lip K.-M., Shen S., Yang X., Keng C.-T., Zhang A., Oh H.-L.J., Li Z.-H., Hwang L.-A., Chou C.-F., Fielding B.C. (2006). Monoclonal antibodies targeting the HR2 domain and the region immediately upstream of the HR2 of the S protein neutralize in vitro infection of severe acute respiratory syndrome coronavirus. J. Virol..

[43] Zhang H., Wang G., Li J., Nie Y., Shi X., Lian G., Wang W., Yin X., Zhao Y., Qu X. (2004). Identification of an antigenic determinant on the S2 domain of the severe acute respiratory syndrome coronavirus spike glycoprotein capable of inducing neutralizing antibodies. J. Virol..

[44] Wang Z., Yuan Z., Matsumoto M., Hengge U.R., Chang Y.-F. (2005). Immune responses with DNA vaccines encoded different gene fragments of severe acute respiratory syndrome coronavirus in BALB/c mice. Biochem. Biophys. Res. Commun..

[45] Yao Y., Jeyanathan M., Haddadi S., Barra N.G., Vaseghi-Shanjani M., Damjanovic D., Lai R., Afkhami S., Chen Y., Dvorkin-Gheva A. (2018). Induction of autonomous memory alveolar macrophages requires T cell help and is critical to trained immunity. Cell.

[46] Widge A.T., Rouphael N.G., Jackson L.A., Anderson E.J., Roberts P.C., Makhene M., Chappell J.D., Denison M.R., Stevens L.J., Pruijssers A.J. (2021). Durability of responses after SARS-CoV-2 mRNA-1273 vaccination. N. Engl. J. Med..

[47] Sahin U., Muik A., Derhovanessian E., Vogler I., Kranz L.M., Vormehr M., Baum A., Pascal K., Quandt J., Maurus D. (2020). COVID-19 vaccine BNT162b1 elicits human antibody and TH 1 T cell responses. Nature.

[48] Paik D.H., Farber D.L. (2021). Anti-viral protective capacity of tissue resident memory T cells. Curr. Opin. Virol..

[49] Mao T., Israelow B., Suberi A., Zhou L., Reschke M., Peña-Hernández M.A., Dong H., Homer R.J., Saltzman W.M., Iwasaki A. (2022). Unadjuvanted intranasal spike vaccine booster elicits robust protective mucosal immunity against sarbecoviruses. bioRxiv.

[50] Knezevic I., Mattiuzzo G., Page M., Minor P., Griffiths E., Nuebling M., Moorthy V. (2021). WHO International Standard for evaluation of the antibody response to COVID-19 vaccines: Call for urgent action by the scientific community. Lancet Microbe.

[51] Omicron-Targeted Vaccines do no Better Than Original Jabs in Early Tests. https://www.nature.com/articles/d41586-022-00003-y.

[52] O’Neill L.A.J., Netea M.G. (2020). BCG-induced trained immunity: Can it offer protection against COVID-19?. Nat. Rev. Immunol..

[53] Ripperger T.J., Uhrlaub J.L., Watanabe M., Wong R., Castaneda Y., Pizzato H.A., Thompson M.R., Bradshaw C., Weinkauf C.C., Bime C. (2020). Orthogonal SARS-CoV-2 serological assays enable surveillance of low-prevalence communities and reveal durable humoral immunity. Immunity.

[54] Doria-Rose N., Suthar M.S., Makowski M., O’Connell S., McDermott A.B., Flach B., Ledgerwood J.E., Mascola J.R., Graham B.S., Lin B.C. (2021). Antibody persistence through 6 months after the second dose of mRNA-1273 vaccine for Covid-19. N. Engl. J. Med..

[55] Andrews N., Stowe J., Kirsebom F., Toffa S., Rickeard T., Gallagher E., Gower C., Kall M., Groves N., O’Connell A.-M. (2022). Covid-19 vaccine effectiveness against the Omicron (B. 1.1. 529) variant. N. Engl. J. Med..

[56] Voysey M., Clemens S.A.C., Madhi S.A., Weckx L.Y., Folegatti P.M., Aley P.K., Angus B., Baillie V.L., Barnabas S.L., Bhorat Q.E. (2021). Single-dose administration and the influence of the timing of the booster dose on immunogenicity and efficacy of ChAdOx1 nCoV-19 (AZD1222) vaccine: A pooled analysis of four randomised trials. Lancet.

[57] Dufloo J., Grzelak L., Staropoli I., Madec Y., Tondeur L., Anna F., Pelleau S., Wiedemann A., Planchais C., Buchrieser J. (2021). Asymptomatic and symptomatic SARS-CoV-2 infections elicit polyfunctional antibodies. Cell Rep. Med..

[58] Khoury D.S., Cromer D., Reynaldi A., Schlub T.E., Wheatley A.K., Juno J.A., Subbarao K., Kent S.J., Triccas J.A., Davenport M.P. (2021). Neutralizing antibody levels are highly predictive of immune protection from symptomatic SARS-CoV-2 infection. Nat. Med..

[59] Mustapha J.O., Abdullahi I.N., Ajagbe O.O., Emeribe A.U., Fasogbon S.A., Onoja S.O., Ugwu C.E., Umeozuru C.M., Ajayi F.O., Tanko W.N. (2021). Understanding the implications of SARS-CoV-2 re-infections on immune response milieu, laboratory tests and control measures against COVID-19. Heliyon.

[60] Kaneko N., Kuo H.-H., Boucau J., Farmer J.R., Allard-Chamard H., Mahajan V.S., Piechocka-Trocha A., Lefteri K., Osborn M., Bals J. (2020). Loss of Bcl-6-expressing T follicular helper cells and germinal centers in COVID-19. Cell.

[61] Hasan A., Al-Ozairi E., Al-Baqsumi Z., Ahmad R., Al-Mulla F. (2021). Cellular and Humoral Immune Responses in Covid-19 and Immunotherapeutic Approaches. ImmunoTargets Ther..

[62] Camou F., Tinevez C., Beguet-Yachine M., Bellecave P., Ratiarison D., Tumiotto C., Lafarge X., Guisset O., Mourissoux G., Lafon M.E. (2021). Feasibility of convalescent plasma therapy in severe COVID-19 patients with persistent SARS-CoV-2 viremia. J. Med. Virol..

[63] Moubarak M., Kasozi K.I., Hetta H.F., Shaheen H.M., Rauf A., Al-Kuraishy H.M., Qusti S., Alshammari E.M., Ayikobua E.T., Ssempijja F. (2021). The rise of SARS-CoV-2 variants and the role of convalescent plasma therapy for management of infections. Life.

[64] Alghamdi A.N., Abdel-Moneim A.S. (2020). Convalescent Plasma: A Potential Life-Saving Therapy for Coronavirus Disease 2019 (COVID-19). Front. Public Health.

[65] Janiaud P., Axfors C., Schmitt A.M., Gloy V., Ebrahimi F., Hepprich M., Smith E.R., Haber N.A., Khanna N., Moher D. (2021). Association of Convalescent Plasma Treatment With Clinical Outcomes in Patients With COVID-19: A Systematic Review and Meta-analysis. JAMA.

[66] Klein S.L., Jedlicka A., Pekosz A. (2010). The Xs and Y of immune responses to viral vaccines. Lancet Infect. Dis..

[67] Klein S.L., Flanagan K.L. (2016). Sex differences in immune responses. Nat. Rev. Immunol..

[68] Conti P., Younes A. (2020). Coronavirus COV-19/SARS-CoV-2 affects women less than men: Clinical response to viral infection. J. Biol. Regul. Homeost. Agents.

[69] Schurz H., Salie M., Tromp G., Hoal E.G., Kinnear C.J., Möller M. (2019). The X chromosome and sex-specific effects in infectious disease susceptibility. Hum. Genom..

[70] Schwarz T., Tober-Lau P., Hillus D., Helbig E.T., Lippert L.J., Thibeault C., Koch W., Landgraf I., Michel J., Bergfeld L. (2021). Delayed antibody and T-cell response to BNT162b2 vaccination in the elderly, Germany. Emerg. Infect. Dis..

[71] Pannus P., Neven K.Y., De Craeye S., Heyndrickx L., Kerckhove S.V., Georges D., Michiels J., Francotte A., Van Den Bulcke M., Zrein M. (2021). Poor antibody response to BioNTech/Pfizer COVID-19 vaccination in SARS-CoV-2 naïve residents of nursing homes. medRxiv.

[72] Castelo-Branco C., Soveral I. (2014). The immune system and aging: A Review. Gynecol. Endocrinol..

[73] Fink A.L., Klein S.L. (2015). Sex and gender impact immune responses to vaccines among the elderly. Physiology.

[74] Wang C.-S., Wang S.-T., Chou P. (2002). Efficacy and cost-effectiveness of influenza vaccination of the elderly in a densely populated and unvaccinated community. Vaccine.

[75] Callaway E. (2021). COVID vaccine boosters: The most important questions. Nature.

[76] Kamar N., Abravanel F., Marion O., Couat C., Izopet J., Del Bello A. (2021). Three doses of an mRNA Covid-19 vaccine in solid-organ transplant recipients. N. Engl. J. Med..

[77] Bar-On Y.M., Goldberg Y., Mandel M., Bodenheimer O., Freedman L., Kalkstein N., Mizrahi B., Alroy-Preis S., Ash N., Milo R. (2021). Protection of BNT162b2 Vaccine Booster against Covid-19 in Israel. N. Engl. J. Med..

[78] Bruxvoort K., Sy L.S., Qian L., Ackerson B.K., Luo Y., Lee G.S., Tian Y., Florea A., Takhar H.S., Tubert J.E. (2022). Real-world effectiveness of the mRNA-1273 vaccine against COVID-19: Interim results from a prospective observational cohort study. Lancet Reg. Health Am..

[79] Thompson M.G., Stenehjem E., Grannis S., Ball S.W., Naleway A.L., Ong T.C., DeSilva M.B., Natarajan K., Bozio C.H., Lewis N. (2021). Effectiveness of COVID-19 vaccines in ambulatory and inpatient care settings. New Engl. J. Med..

[80] Chung J.Y., Thone M.N., Kwon Y.J. (2020). COVID-19 vaccines: The status and perspectives in delivery points of view. Adv. Drug Deliv. Rev..

[81] Pati R., Shevtsov M., Sonawane A. (2018). Nanoparticle vaccines against infectious diseases. Front. Immunol..

[82] Chauhan G., Madou M.J., Kalra S., Chopra V., Ghosh D., Martinez-Chapa S.O. (2020). Nanotechnology for COVID-19: Therapeutics and vaccine research. ACS Nano.

[83] Sepand M.R., Aghsami M., Keshvadi M.H., Bigdelou B., Behzad R., Zanganeh S., Shadboorestan A. (2021). The role of macrophage polarization and function in environmental toxicant-induced cancers. Environ. Res..

[84] Sepand M.R., Ghavami M., Zanganeh S., Stacks S., Ghasemi F., Montazeri H., Corbo C., Derakhshankhah H., Ostad S.N., Ghahremani M.H. (2020). Impact of plasma concentration of transferrin on targeting capacity of nanoparticles. Nanoscale.

